# Structural relationships and vasorelaxant activity of monoterpenes

**DOI:** 10.1186/2008-2231-20-23

**Published:** 2012-09-03

**Authors:** Tamires Cardoso Lima, Marcelo Mendonça Mota, José Maria Barbosa-Filho, Márcio Roberto Viana Dos Santos, Damião Pergentino De Sousa

**Affiliations:** 1 Department of Physiology, Federal University of Sergipe, CEP 49100-000, São Cristóvão, Sergipe, Brazil; 2 Laboratório de Tecnologia Farmacêutica, Federal University of Paraíba, Caixa Postal 5009, CEP 58051-970, João Pessoa, Paraíba, Brazil

**Keywords:** Essential oils, Terpenes, Spasmolytics, Structure-activity relationships, Cardiovascular activity

## Abstract

**Background and purpose of the study:**

The hypotensive activity of the essential oil of *Mentha* x *villosa* and its main constituent, the monoterpene rotundifolone, have been reported. Therefore, our objective was to evaluate the vasorelaxant effect of monoterpenes found in medicinal plants and establish the structure-activity relationship of rotundifolone and its structural analogues on the rat superior mesenteric artery.

**Methods:**

Contractions of the vessels were induced with 10 μM of phenylephine (Phe) in rings with endothelium. During the tonic phase of the contraction, the monoterpenes (10^-8^ - 10^-3^, cumulatively) were added to the organ bath. The extent of relaxation was expressed as the percentage of Phe-induced contraction.

**Results:**

The results from the present study showed that both oxygenated terpenes (rotundifolone, (+)-limonene epoxide, pulegone epoxide, carvone epoxide, and (+)-pulegone) and non-oxygenated terpene ((+)-limonene) exhibit relaxation activity. The absence of an oxygenated molecular structure was not a critical requirement for the molecule to be bioactive. Also it was found that the position of ketone and epoxide groups in the monoterpene structures influence the vasorelaxant potency and efficacy.

**Major conclusion:**

The results suggest that the presence of functional groups in the chemical structure of rotundifolone is not essential for its vasorelaxant activity.

## Introduction

The essential oils are natural products extracted from species of aromatic plants and exhibit a variety of biological properties [1-6]. These effects are attributed mainly to the terpenes, which are the major chemical components of these oils. The cardiovascular activity of essential oils has been reported [7-10]. Some studies showed that the main chemical components of these oils are bioactive [7,10-12].

The antihypertensive activity of some plants also has been related to the presence of terpenes. Extracts and diterpenoids of *Salvia* species have shown cardiovascular activity, such as ferruginol and 7-oxo-abieta-9,12,14-triene [13]. In another work, in normotensive anaesthetised rats, the cardiovascular effect of the essential oil of *Mentha* x *villosa* and its main constituent, rotundifolone, suggests that hypotensive activity may result from its vasodilatory effects directly upon vascular smooth muscle [11]. Additional studies showed that the hypotensive activity of the monoterpene rotundifolone is related to decrease in heart rate and peripheral vascular resistance, probably via non-selective muscarinic receptor stimulation [14].

For these reasons, it appeared possible that terpenes found in essential oils could also have cardiovascular activity. Therefore, our objective was to verify the potential vasorelaxant effect of representative monoterpenes present in medicinal plants and to determine the relationship between the chemical structure of rotundifolone and its vasorelaxant activity to understand the influence of the functional groups present in this monoterpene. Therefore, we assessed *p*-menthane monoterpenes containing ketone groups and/or epoxy in different positions in the chemical structure. In addition, a monoterpene that does not contain these functional groups was also evaluated.

## Materials and methods

### Chemicals and solutions

(+)-Limonene epoxide was prepared via oxidation of (+)-limonene with m-chloroperoxybenzoic acid [15]. Pulegone epoxide [16] and carvone epoxide [17] were prepared via oxidation of (+)-pulegone and (−)-carvone, respectively, using hydrogen peroxide in alkaline medium as previously described. (+)-Pulegone was purchased from Aldrich. (+)-Limonene was purchased from company Dierberger Óleos Essenciais S.A., Barra Bonita, Brazil. Rotundifolone was isolated from the essential oil of *Mentha* x *villosa* using a previously described procedure [18]. The purity of test compounds was higher than 95%. L-Phenylephrine chloride (Phe) and acetylcholine chloride (Ach) were purchase from Sigma (Sigma Chemical Co., USA). In the preparation of the stock solutions, the monoterpenes were diluted in Tyrode/cremophor (0.15% v/v) solution. Phe and Ach were diluted or in Tyrode solutions only. All stock solutions were maintained at 0°C and diluted to the desired concentration, when necessary. Cremophor (Sigma Chemical Co., USA) in used concentrations showed no effect on control experiments (data not shown).

(Composition of Tyrode’s solution in mM: NaCl 158.3, KCl 4.0, CaCl_2_ 2.0, NaHCO_3_ 10.0, C_6_H_12_O_6_ 5.6, MgCl_2_ 1.05 and NaH_2_PO_4_ 0.42).

### Animals

Male Wistar normotensive rats (200 – 300 g) were obtained from colonies maintained in the Department of Physiology, Federal University of Sergipe, Sergipe, Brazil. They were maintained in a large cage under controlled conditions of temperature and lighting (lights on: 06:00–18:00 h), fed with rodent diet and tap water *ad libtum*. All procedures were approved by the Animal Research Ethics Committee of the Universidade Federal de Sergipe, Brazil (Protocol number 15/2009) and were in compliance with the Guide for the Care and Use of Laboratory Animals published by the US National Institutes of Health (NIH publication 85–23, revised 1996).

### Pharmacological assays

The tissue preparation was performed as described in Menezes et al. [19]. Rats were sacrificed by exsanguination under ether aesthesia and superior mesenteric artery was removed, cleaned from connective and fat tissues and sectioned in rings (1 – 2 mm). These rings were suspended in organ baths containing 10 mL of Tyrode’s solution, gassed with carbogen and maintained at 37°C under a resting tension of 0.75 g for 60 min (stabilization period). The isometric tension was recorded through a force transducer (Letica, Model TRI210, Italy) coupled to an amplifier-recorder (AVS, Brazil). Endothelium functionality of the rings was assessed by the ability of Ach (10 μM) to induce more than 70% relaxation of Phe (10 μM) tonus.

After verify the functionality of vascular endothelium, contractions of the vessels were induced with 10 μM Phe in rings with endothelium. During the tonic phase of the contraction, the monoterpenes rotundifolone, (+)-limonene epoxide, pulegone epoxide, carvone epoxide, (+)-pulegone and (+)-limonene (10^-8^ - 10^-3^, cumulatively) were added to the organ bath. The extent of relaxation was expressed as the percentage of Phe-induced contraction.

### Statistic analysis

Values were expressed as the mean ± SEM. The results were analyzed with one-way ANOVA followed by Bonferroni post-test. The potency was expressed as pD_2_ value (negative logarithm of molar concentration producing the half maximum effect - E_max_) of *in vitro* experiments were obtained by non-linear regression. All procedures were performed by using Graph Pad Prism 3.02™.

## Results and discussion

**Figure 1:**
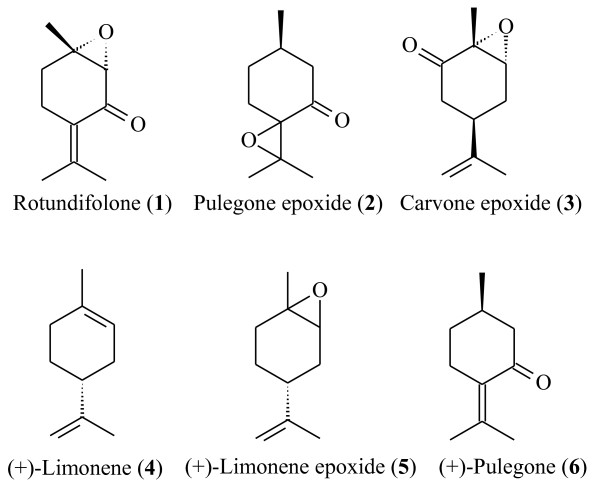
Chemical structures of the monoterpenes used in this study.

Several studies have reported pharmacological effects of essential oils on the rat cardiovascular system [9-11,20,21]. However, there are few studies on bioactive compounds that contribute to the pharmacological activity of these oils. We report in this comparative study the findings on the vasorelaxant activity of six monoterpenes (Figure1): rotundifolone (having α,β-unsaturated ketone and endocyclic epoxide groups), limonene epoxide (having only an epoxide group), pulegone epoxide (having ketone and exocyclic epoxide groups), carvone epoxide (having ketone and endocyclic epoxide groups), (+)-pulegone (having an α,β-unsaturated ketone), and (+)-limonene (non-oxygenated terpene).

The investigation of the influence of the position of ketone and epoxide groups in the molecular structure was performed by comparing the rotundifolone (**1**) with the pulegone epoxide (**2**) and carvone epoxide (**3**). The rotundifolone showed a vasorelaxant effect in rat mesenteric artery more potent than carvone epoxide (*p* < 0.01), suggesting that the position of the ketone group influences this spasmolytic property. In addition, the pharmacological activity of rotundifolone was lower than the pulegone epoxide (*p* < 0.05). This result demonstrates that the position of epoxide group in the *p*-menthane structure alters the pharmacological activity. The experimental data also showed that the pulegone epoxide exhibit more significant vasorelaxant activity in comparison to carvone epoxide (*p* < 0.001), indicating that the exocyclic epoxide group contributes more to the pharmacological effect than the endocyclic epoxide group. These results confirm that the position of epoxide and ketone functional groups in molecules change the vasorelaxant activity. Comparing rotundifolone with (+)-limonene (**4**), these compounds showed equipotent vasorelaxant activity (*p* > 0.05). The spasmolytic activity of (+)-limonene shown in this study demonstrates that non-oxygenated terpenes present in cardioactive essential oils may contribute to this activity. (+)-Limonene epoxide (**5**) and (+)-pulegone (**6**) presented vasorelaxant activity below 50%, so it was not allowed to calculate their pD_2_ (Table1).

**Table 1:** **Values of pD**_**2 **_**and E**_**max **_**obtained of concentration-response curves to monoterpenes in isolated rings of rat superior mesenteric artery with functional endothelium pre-contracted with Phe (10 μM)**

**Monoterpenes**	**N**	**pD**_**2**_	**E**_**max**_
Rotundifolone (**1**)	4	5.1 ± 0.2	71.7 ± 6.2
Pulegone epoxide (**2**)	4	6.3 ± 0.3^a,c^	55.5 ± 3.0
Carvone epoxide (**3**)	4	3.4 ± 0.3^e^	99.8 ± 18.7**^,^***
(+)-Limonene (**4**)	4	5.1 ± 0.1	84.2 ± 4.1^##^
(+)-Limonene epoxide (**5**)	4	---	34.0 ± 2.0
(+)-Pulegone (**6**)	4	---	30.3 ± 2.3*

N = Number of experiments; pD_2_ – Potency; E_max_ – Maximum Effect; ^a^*p* < 0.05 *vs* rotundifolone and (+)-limonene; ^c^*p* < 0.001 carvone epoxide, and ^e^*p* < 0.01 *vs* rotundifolone and (+)-limonene. *p < 0.05 vs rotundifolone, ***p < 0.001 *vs* (+)-pulegone and limonene epoxide; **p < 0.01 *vs* pulegone epoxide, and ^##^*p* < 0.01 vs (+)-pulegone and limonene epoxide.

Similarly, the effectiveness of the tested compounds was investigated by comparing their maximum effects. The rotundifolone showed a higher pharmacological effect than (+)-pulegone (*p* <0.05). While (+)-limonene was more effective than (+)-pulegone (*p* <0.01) and limonene epoxide (*p* <0.01). Carvone epoxide exhibited a maximum effect of relaxation greater than pulegone epoxide (*p* <0.01), (+)-pulegone (*p* <0.001), and limonene epoxide (*p* <0.001).

It is described in the literature that the monoterpene terpinen-4-ol produces hypotensive effect in rats. Terpinen-4-ol is the main constituent of the essential oil of *Alpinia zerumbet* (Pers.) B. L. Burtt. & R. M. Sm*.* (Zingiberaceae). Lahlou *et al.*[12] proved that the hypotensive effects of the essential oil of *Alpinia zerumbet* are partially attributed to the actions of terpinen-4-ol, which was more potent than this oil. In another study, Barbosa-Filho *et al*. [22] showed that the essential oils from stem and root of the plant *Ocotea duckei* have hypotensive activity. The main chemical constituents of these oils are the terpene β-eudesmol and elemol, respectively. These reports, together with our findings, demonstrate the importance of this natural chemical class as good candidates for antihypertensive drugs.

Furthermore, studies have demonstrated that some of these terpenes exhibit depressant properties in other organic systems. Sadraei *et al*. [4] and Camara *et al*. [5] showed that both, (+)-α- and (−)-β-pinene, relax rat and guinea-pig ileum, respectively. Other studies have shown that (±)-linalool promotes spasmolytic effect in guinea-pig ileum mediated by cAMP [23], and recently Tanida *et al*. [24] demonstrated that the inhalation of (±)-linalool reduces the blood pressure mediated by the central nervous system in rats. Therefore, the hypotensive activity could originate from vasodilatory effects induced by these compounds. This possible mechanism of action was studied in citronellol, which lowers blood pressure by a direct effect on the vascular smooth muscle leading to vasodilation [25].

The biotransformation of some compounds tested is known. For example, (+)-limonene is biotransformed to several metabolites, such as (+)-limonene epoxide, carvone, and perillyl alcohol [26,27]. (+)-Limonene epoxide is one of the bioactive compounds of the present study. Carvone has ketone group which is present in the chemical structure of carvone epoxide, another evaluated compound. The monoterpene perillyl alcohol has hypotensive activity [28]. Pulegone also produces bioactive metabolites, such as menthol [29] which has vasorelaxant activity [30]. Therefore, the metabolites of (+)-limonene, pulegone, and other tested compounds should contribute to their vasorelaxant activity. In addition, the tested compounds must act by different mechanisms of action. In fact, monoterpene alcohols, aldehydes, ethers, and hydrocarbons show effects on the cardiovascular system via vasorelaxation, decreased heart rate or hypotension, among others [31]. The mechanisms of action of these compounds should be related to their metabolites.

## Conclusion

The results from the present study showed that both oxygenated terpenes and hydrocarbon terpene exhibit spasmolytic activity. The absence of an oxygenated molecular structure was not a critical requirement for the molecule to be bioactive. The position of ketone and epoxide groups in the *p*-menthane structure influences the vasorelaxant potency and efficacy. Therefore, these functional groups contribute to the vasorelaxant activity of rotundifolone.

## Competing interests

The authors declare that they have no competing interests.

## Authors’ contributions

TCL was responsible for the preparation of monoterpenes and wrote the chemical content of the manuscript; MMM evaluated the pharmacological activity of monoterpenes; JMB-F isolated and identified the rotundifolone; MRVDS interpreted and wrote the pharmacological data of the manuscript; DPDS analyzed, interpreted and corrected the structure-activity relationships data of monoterpenes. All authors read and approved the final manuscript.
